# Impact of particle size, temperature and humic acid on sorption of uranium in agricultural soils of Punjab

**DOI:** 10.1186/s40064-015-1051-2

**Published:** 2015-06-17

**Authors:** Ajay Kumar, Sabyasachi Rout, Manish Kumar Mishra, Rupali Karpe, Pazhayath Mana Ravi, Raj Mangal Tripathi

**Affiliations:** Health Physics Division, Bhabha Atomic Research Centre, Trombay, Mumbai, India

**Keywords:** Uranium, Soil, Sorption, Particle size, Temperature, HA

## Abstract

Batch experiments were conducted to study the sorption of uranium (U) onto soil in deionised water as a function of its dosage, temperature and humic acid (HA). Furthermore, soils were characterized for particle sizes in the form of sand (>63 µm), silt (>2–<63 µm) and clay (<2 µm). The textural analysis revealed that soils were admixture of mainly sand and silt along with a small abundance of clay. X-ray diffraction analysis indicates that clay factions ranging from 2.8 to 5% dominated by quartz and montmorillonite. Experimental results indicated that soil with high abundance of clays and low sand content has relatively high U sorption which could be due to availability of high exchange surfaces for metal ions. However, at low concentration of HA, sorption of U was maximum and thereby decreased as the HA concentration increased. The maximum sorption may be due to increase in the negative active surface sites on HA and further decrease could be attributed to saturation of sorption site and surface precipitation. Conversely, the thermodynamic data suggested that the sorption is spontaneous and enhanced at higher temperature.

## Introduction

During the past decades, agricultural activities in Punjab widely expanded causing an escalation in the application of inorganic fertilizers, pesticides and other agricultural chemicals to increase crop production and to enhance soil properties. The contaminants accumulation in soil due to long-continued agricultural activities will depend on its concentrations in fertilizers, annual application rate of fertilizers, physical and chemical properties of soil and geochemical properties of the contaminant itself. The distribution of U in soil are generally influenced by sorption, complexation processes on inorganic soil constituents such as clay minerals, oxides and hydroxides (silica, aluminium, iron and manganese), biological fixation and transformation of organic matter (Belivermis et al. 2009; Bolivar et al. 1995). The abundances of radionuclides and their occurrences in the environment are a result of anthropogenic activities as well as natural processes (Bolivar et al. 1995). The migration of U through soil is enhanced by rainwater (precipitation) and greatest in areas with heavy rainfall. Since the textural and mineralogical information of soils is also essential for understanding soil genesis and for developing appropriate management practices in the maintenance of soil fertility (Marsonia et al. 2008). Therefore, attempts have been made to study the textural and mineralogical characteristics of agricultural soils in the uranium sorption studies. Uranium (VI) forms very stable carbonato complexes in solution and as a consequence uranium sorption in the presence of dissolved CO_2_ is strongly suppressed in comparison to the carbonate free system (Kowal-Fouchard et al. 2004; Katsoyiannis 2007; Dong et al. 2005; Alliot et al. 2005; Hartmann et al. 2008). Due to very low solubility of the tetravalent uranium [U(IV)], it has a strong tendency toward hydrolysis under relevant natural aquatic system conditions (Choppin 2006). This leads to a strong interaction (sorption) with any kind of surfaces, even at low pH (Clark et al. 2011; Landa et al. 1995; Murphy et al. 1999). Precipitation or polymer/colloid formation due to oversaturation (Dähn et al. 2002; Carroll et al. 1992; Neck et al. 2003) has to be expected as side reactions in sorption studies of tetravalent uranium.

Furthermore, natural organic matters (NOMs) present in soil also play an important role in the fate and transport behaviour of uranium in which they form strong complexes, which are affected by the extent of organic interaction with mineral surfaces and thereby depends on pH. The sorption of uranium onto mineral surfaces in the presence of humic substances had been reported by many researchers (Schmeide et al. 1999; Pompe et al. 1999). There are a large number of possible reactions and interactions of uranium with OM which depends on the pH of the soil, the cation concentration in the soil, the functional group and the degree of saturation of the potential sorption sites.

Studies on the effect of temperature and uranium concentration on the sorption of uranium to a number of pure minerals were conducted where idealized distribution coefficients (k_d_) are calculated from Freundlich isotherms (Langmuir 1978; Syed 1999; Choppin 2007; His and Langmuir 1985). In the present study, besides the textural and mineralogical characteristics of soils, the sorption of uranium was examined as a function of its concentrations, temperature and HA using batch experiment techniques.

## Materials and methods

### Sampling sites

A total of 8 representative agricultural surface soil samples (a depth range of 5–30 cm) were collected from Bathinda district in Punjab in the month of March, 2014. The sampling was done using an auger soil sampler, stored in polyethylene bags and transported to the laboratory. The geographical location of the sampling area is south-west of Punjab between latitude 29°07′N–30°57′N and longitude 74°05E−76°55′E at an average elevation of 200 m from the sea level. Average annual rainfall is 500 mm of which 80% is received during the period of June–October. The soil of the study area is loose, sandy, calcareous and alluvial, which is an admixture of gravel, sand, silt and clay in various proportions.

### Soil sampling and pre-treatment

The collected soil samples were dried at 110°C for 24 h, powdered, homogenized and sieved through 110 mesh sizes. The powdered samples were thoroughly mixed with each other and prepared for two sets (Set-1 and Set-2). Each set was washed thrice with deionised water. The solid phase was allowed to settle by centrifugation and the washing solution was discarded. After washing, samples were further dried at 110°C, placed in conical flasks and stored as stock samples for experimental work.

### Sorption studies

A batch equilibrium experiment was conducted to determine the sorption of U in terms of percentage (%) which is given by the Eq. (1) (Bachmaf and Merkel 2011; Kumar et al. 2012) and in terms of k_d_ using Eq. (2) (Kumar et al. 2012; Rout et al. 2014):

1 $$q_{e} = \left[ {\frac{{C_{i} - C_{e} }}{{C_{i} }}} \right] \times 100$$

2 $$k_{d} = \left( {\frac{{C_{i} - C_{e} }}{{C_{e} }}} \right)\; \times \;\frac{V}{m}$$

where C_i_ is the initial concentration of U in the solution; C_e_ is the final concentration in solution after reaching equilibrium, V is the volume of the contact solution and m is the mass of the soil. In the present study, 5 g dried agricultural soil samples of each set were placed in each of eight empty PTFE (Poly Tetra Fluoro Ethylene) containers with lid to avoid significant sorption and equilibrated for 7 days with 150 mL of deionised water containing 1 mgL^−1^ (Batch-1), 3 mgL^−1^ (Batch-2), 5 mgL^−1^ (Batch-3), 7 mgL^−1^ (Batch-4), 10 mgL^−1^ (Batch-5), 20 mgL^−1^ (Batch-6), 30 mgL^−1^ (Batch-7) and 40 mgL^−1^ (Batch-8) of U standard [UO_2_ (NO_3_)_2_∙6H_2_O] followed by shaking using end-over end shaker (model: 300, Korea make) at 298 K. After equilibration time, the samples of each batch were centrifuged, filtered through 0.45 µm filter paper and supernatant analyzed for U.

In the similar fashion, a batch experiment was also conducted to determine the k_d_ values of U in both sets of soil as a function of HA concentrations (2.5, 5, 10, 25, 50, 100 and 125 mgL^−1^) spiked with 1 mgL^−1^ of U standard. The pH of the equilibrated solution was maintained within the range of 5.5–6 throughout all experiments after addition of desired amount of 0.1 M of NaOH or 0.1 M HNO_3_ (Merck, Mumbai, India) using an automated titrator (Metrohm-798 MPT Titrino, Switzerland) in “pH–stat” mode. Blank samples were also run in absence of soils at different HA concentrations. Duplicate samples of each soil and one experimental blank were also analyzed and served as an internal check on the precision of the analytical results.

### Uranium estimation

The concentration of U in aliquots of equilibrium solution was measured by uranium analyser UA-2 (Quantalase, Indore, India) in which LED (Light Emission Diode) is used to excite uranyl species present in the sample, which on de-excitation gives out fluorescence peak. Finally standard addition technique was followed for the estimation of U in the samples. The instrument was calibrated in the range of 1–100 μgL^−1^ using a stock solution of (1 gL^−1^) UO_2_ (NO_3_)_2_∙6H_2_O standard (USA). 5% sodium pyrophosphate in ultra pure water was used as fluorescence reagent (Kumar et al. 2014a). All the experimental data were the averages of duplicate or triplicate experiments. The relative standard deviation (RSD) was calculated to be 5–8%. Quality assurance was carried out by spike recovery, replicate analysis and cross method checking.

### Thermodynamics studies

k_d_ values of U in soil (set-2) for Batch-1, Batch -3 and Batch-5 were obtained at three particular temperatures such as 298, 323 and 348 K (Sheng et al. 2012) which were maintained using an incubator-cum shaker. Subsequently, the thermodynamic parameters (∆H°, ∆S° and ∆G°) for U sorption onto soil were obtained at temperature dependent isotherm. The values of standard enthalpy change (∆H°) and standard entropy change (∆S°) were calculated from the slope and y-intercept of the plot of lnk_d_ versus 1/T using Eq. (3): (Sheng et al. 2012; Kumar et al. 2013),

3 $$\ln k_{d} = \frac{\varDelta S}{R} - \frac{\varDelta H^\circ }{RT}$$

Similarly, values of the standard free energy (∆G°) were calculated using Eq. (4)

4 $$\varDelta G^\circ = \varDelta H^\circ - T\varDelta S^\circ$$

where R is the universal ideal gas constant (8.314 Jmol^−1^K^−1^), T is the temperature in Kelvin.

### XRD analysis

The mineralogical study of soil samples were also carried out using X-ray diffractometer (XRD, Model: GNR, Italy). The XRD data were collected on an APD-2000 diffractometer equipped with a 6-position sample holder, theta–theta goniometer and a NaI (Tl) scintillation detector. A Ni-filtered CuK_ɑ_ radiation (λ = 0.154 nm) at applied voltage of 40 kV and current of 30 mA was used. For phase identification, Search and Match procedure was performed by using GNR’s SAX software with ICDD Reference Database.

### Particle size distribution

The particle size distribution of soil samples was determined using a laser diffraction particle size analyzer (CILAS, France, Model 1190). For soil texture analysis, three different laser diffraction methods identified as LDM 1, LDM 2 and LDM 3 were considered. In LDM 1, the soil sample is thoroughly mixed before analysis. In LDM 2, the sand fraction is sieved out and analyzed separately from the silt–clay fraction. LDM 3 is similar to LDM 2 except that the silt–clay fraction is diluted so that a large sample volume can be used while maintaining an acceptable level of obscuration. LDM 2 and LDM 3 improve the particle size distribution (PSD) in comparison to LDM 1, without the need of altering the Mie theory parameters. Finally, the PSD of the silt–clay and sand were quantified in terms of percent (%) based on the relative weight of each fraction. PSD was performed with a small angle light scattering apparatus equipped with a low-power (2 mW) Helium–Neon laser with a wavelength of 633 nm as the light source. The apparatus has active beam length of 2.4 mm, and it operates in the range 0.04–2,500 lm. The obscuration levels of samples in the laser diffractometry analysis were kept between 15 and 25%. Maintaining this obscuration levels in sediments with high clay contents (20%) compelled to use small volumes because of the high optical density of clay. A 2 g aliquot of the soil sample was introduced into the ultrasonic bath. Finally, the PSD was obtained using two optical models, the Fraunhofer diffraction model and the Mie theory. Because the Fraunhofer model is not accurate enough for the determination of the clay–size fraction. The Mie theory applies rigorously to spherical, homogeneous particles and fits less satisfactorily nonspherical or non homogenous particles as commonly found in sediments. The details of particle size distribution methods are also described in Kumar et al. (2014b).

### Chemical characterization

The total carbon, nitrogen and hydrogen in soil and HA were estimated using C H N S–O elemental analyser (Flash EA 1112 Series, Thermo Finnigan, Italy). The elemental analyzer was calibrated and standardized using BBOT Standard [2, 5-bis (5-tert-butyl-benzoxazol-2-yl)-thiopen, C_26_H_26_N_2_O_2_S, Thermo Finnigan, Italy)]. The minimum detection limit for C, N and H was calculated to be 0.08%. The other elements (K, Ca, Fe, Cu, Ni, Co, Mn and S) in soil were also quantified using Bench top Energy Dispersive X-ray fluorescence technique (EDXRF, Oxford Instrument, X- 5000, Germany). The sample targets were excited using the incident beam from the X-ray tube (10 W long-fine-focus Rh-anode) operated at anode voltage of 50 kV.

## Results and discussions

### Effect of U concentrations

**Figure 1:**
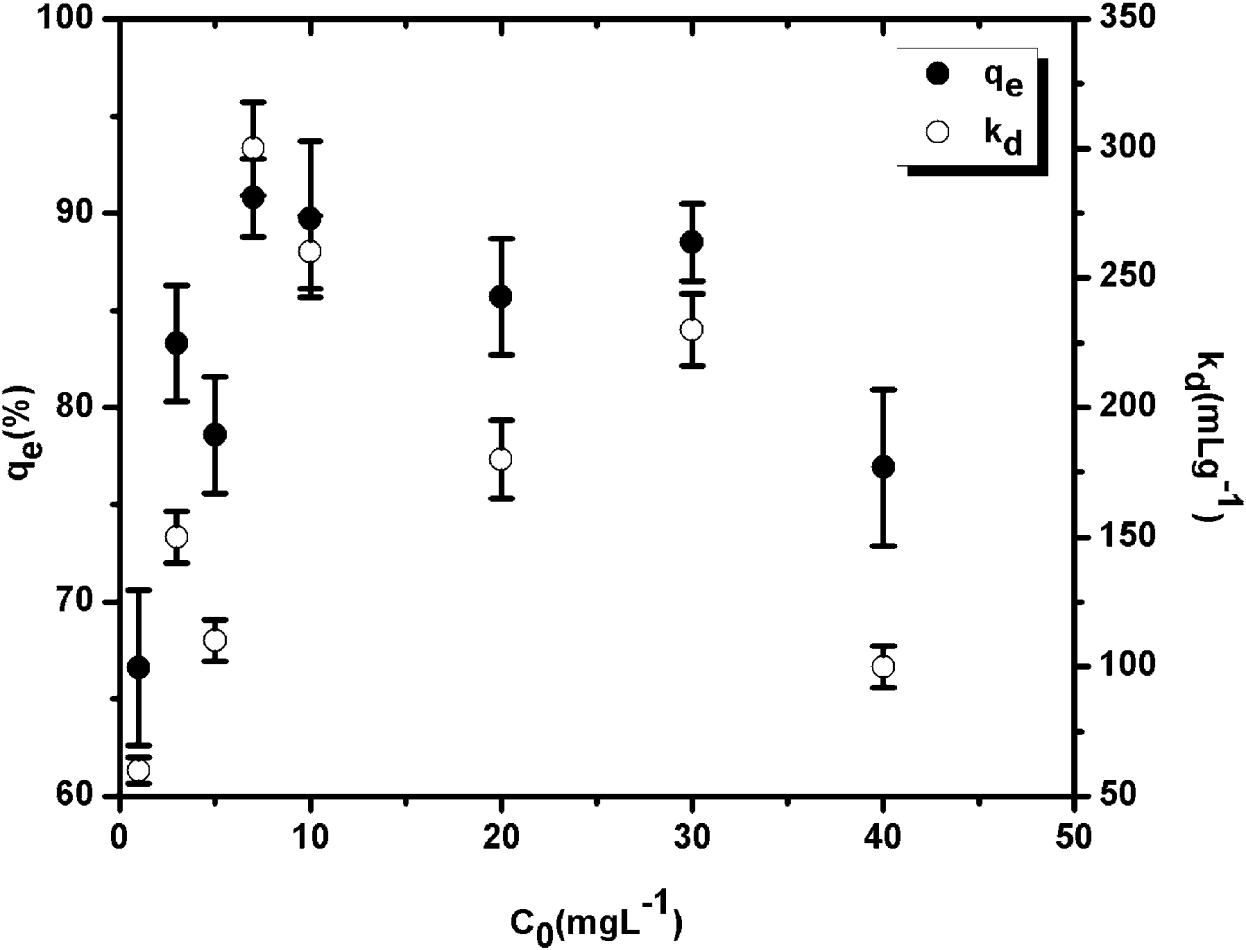
Variation of sorption and k_d_ values as a function of U concentration in soil (set-1).

**Figure 2:**
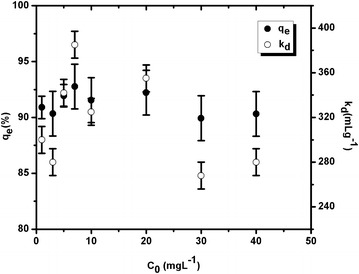
Variation of sorption and k_d_ values as a function of U concentration in soil (set-2).

The sorption of U (VI) onto soils in terms of k_d_ values was initially examined for varying uranium concentrations (1–40 mgL^−1^) at constant temperature under the same laboratory conditions. Figures 1, 2 illustrate the variations of sorption and k_d_ values as a function of U concentrations in agricultural soils. Subsequently, the percentage of sorbed uranium in set-1 and set-2 ranged to be about 67–91% and 89–93% respectively. Similarly the k_d_ values were obtained in the range of 60–300 mLg^−1^ (mean: 174 ± 84 mL g^−1^) and 268–385 mL g^−1^ (mean: 317 ± 42 mL g^−1^) for the same throughout the entire batches. In set-1, initially, at low concentration range (1–7 mgL^−1^), sorption generally increases as U concentration increases; thereafter it decreases at sufficiently high concentration. However, set-2 did not show any significant variation in U sorption as seen by narrow range of percentage sorption. The increasing trend of sorption at low concentration range in set-1 might be due to strong bonding energies of U with the surface functional groups at sorption sites of soil. On the contrary, when the specific bonding sites become increasingly occupied, sorption becomes unspecific at high concentrations, resulting in lower k_d_ values (Alloway 1995; Shaheen et al. 2009; Saha et al. 2002). However, in set-2, an almost uniform sorption of U onto soil observed, which might be caused for reasonably efficient amount of soil, when the optimum U concentration used over 1 mg L^−1^.

### Effect of particle size

In general, the soils were mainly composed of sand and silt. Particle sizes of soils of both sets were characterized as sand (>63 µm), silt (>2–<63 µm) and clay (<2 µm). Soils of set-1 were sandy- silt loam in the form of 54.2% sand, 42% silt and 3.8% clay whereas set-2 were silty-sand with the distribution of 36% sand, 59% silt and 5% clay. The mean diameter of particle size of soils ranged from 53 to 86 µm (Mean: 69.4 ± 11.5 µm) along the studied area. The comparatively higher sorption of U onto soil of set-2 might be due to presence of high amount of finer particles in the form of clays and low sand content. This can be confirmed by obtaining a strong positive correlation between U and clays content in the past study. However, sand and silt did not show any particular significant correlation (Kumar et al. 2015). In literatures, the huge variation observed in k_d_ values of uranium in various soil types (Kaplan et al. 1998; Gamerdinger et al. 1998; USEPA 1999).

### Effect of temperature

**Figure 3:**
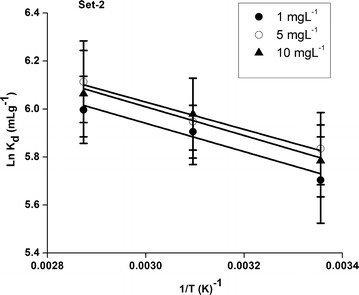
The linear plot of lnk_d_ versus 1/T for U sorption onto soil of set-2, pH = 8.8, C (U)_initial_ = 1, 5, 10 mgL^−1^.

The sorption isotherm in terms of k_d_ in soil of set-2 at three particular spiked U concentration of 1 mg L^−1^ (Batch-1), 5 mg L^−1^ (Batch-3) and 10 mg L^−1^ (Batch-5) was examined at T = 298, 323 and 348 K under the similar laboratory conditions as shown in Figure 3. The measured mean k_d_ values over the three batches at 298, 323 and 348 K was found to be 322 ± 21, 382 ± 14 and 428 ± 25 mLg^−1^ respectively. This clearly indicates that the sorption of U increases as T increases and this process is more pronounced at higher temperature. The elevated sorption at high temperature may be due to increase in diffusion rate of U into the pores of soils (Chen et al. 2009; Zhao et al. 2010; Kumar et al. 2013). Changes in soil pore sizes as well as an increase in the number of active sorption sites due to breaking of some internal bonds near soil surface edge are generally expected at higher temperatures. Therefore, the increase in temperature may result in the increase in the affinity of U to the soil surface (Ghosh et al. 2008).

The determination of the thermodynamic parameters (ΔH°, ΔS° and ΔG°) can provide mechanism insights into U sorption onto soils. The values of ∆H° were positive, which is indicative of an endothermic sorption process. The possible reason for the endothermic process is that U(VI) ions are well solvated in water and at higher temperature, these ions are denuded from their hydration sheath onto soils leading to less hydrated than those in solution. The removal of water molecules from U ions is essentially an endothermic process and the endothermicity of the desolvation process exceeds the enthalpy of sorption to a considerable extent (Hu et al. 2010; Yang et al. 2010, 2011a, b; Kumar et al. 2013). Moreover, the values of ΔG° were all negative at all temperatures studied herein as expected for a spontaneous process under our experimental conditions. The higher the reaction temperature, the more negative the value of ΔG°, indicating that the adsorption reaction is more favorable at elevated temperatures (Hu et al. 2010). At high temperature, U(VI) ions are readily dehydrated and thereby their sorption becomes more favorable. However, the values of ΔS° were all positive indicating that during the whole adsorption process, some structural changes occurs on soils surface leading to an increase in the disorderness at the soil–water interface (Hu et al. 2010). The slightly higher values of ΔS° revealed a more efficient sorption at higher temperature (Zhao et al. 2010; Yang et al. 2009, 2011a, b). Table 1 illustrates values of thermodynamic parameters (∆G°, ∆H° and ∆S°) for the sorption of U onto soil of set-2.

**Table 1:** Thermodynamic parameters for the sorption of U onto soil (set-2)

C_0_ (mg L^−1^)	∆Hº (kJ mol^−1^)	∆Sº (Jmol^−1^K^−1^	∆Gº (kJ mol^−1^)		
			298 K	323 K	348 K
1	4.91	64.10	−14.19	−15.80	−17.39
5	4.74	64.35	−14.44	−16.05	−17.66
10	4.97	64.85	−14.35	−15.97	−17.60

### Effect of HA

**Figure 4:**
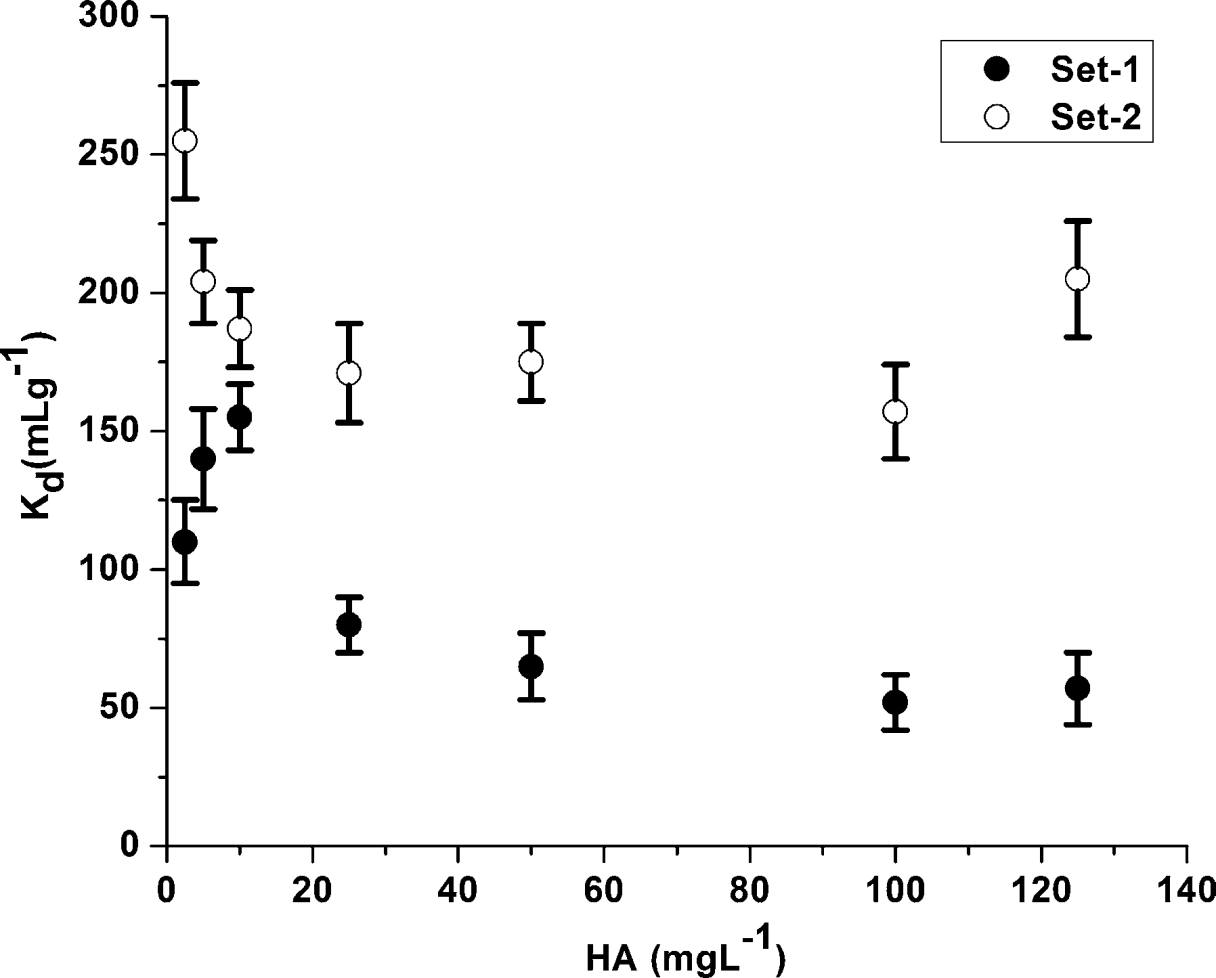
Variation of k_d_ values of uranium in soils as a function of HA concentration.

In presence of high inorganic carbonate concentration, there is little effect of HA on uranium adsorption. These inorganic carbonates with their high complexing ability towards uranyl ions predominate the influence of HA at pH 3.5–9.5. In fact the cationic uranyl ion remaining in the solution can be associated with HA which either is sorbed onto the soil or is dissolved. In the present study, k_d_ values of U as a function of HA concentration for set-1 and set-2 as depicted in Figure 4 were obtained to be in the range of 52–155 L kg^−1^ (mean: 94 ± 41 L kg^−1^) and 157–255 L kg^−1^ (mean: 193 ± 32 L kg^−1^) throughout entire experimental respectively. From figure, it is obvious that initially, in general, at low concentration (2.5–10 mg L^−1^) of HA, k_d_ values of U was found to be relatively higher and thereby decreased as the HA concentration increased. Overall, k_d_ values decreased as HA concentration increases.

Literatures have reported that at acidic pH range (3.5–6), the sorption of U generally increased in the presence of HA which is due to increase in the negative sorption active sites on HA. However, sorption decreases at higher pH (>6), probably due to formation of soluble uranyl humate complexes species (Pompe et al. 1999). As pH increases, the increased deprotonation makes the HA more negatively charged. This negative charge creates an electrical field which influences the complexation reaction. HA concentration must be high enough to influence the uranium adsorption onto minerals due to the competition from other anionic ligands especially in the slightly acidic to alkaline pH range.

In the previous study, FTIR spectra of soils of the studied area were recorded and confirmed a silicate group only (Kumar et al. 2013). The obtained absorption bands for soils were in poor agreement with the HA and thus poorly enriched with respect to organic matters. This is also confirmed by the presence of C content in soils which showed the mean value of 0.89%. Due to low abundances of carbon, soils of set 1 might have shown poor sorption with U leading to relatively lower k_d_ values.

### XRD spectra of soil

**Figure 5:**
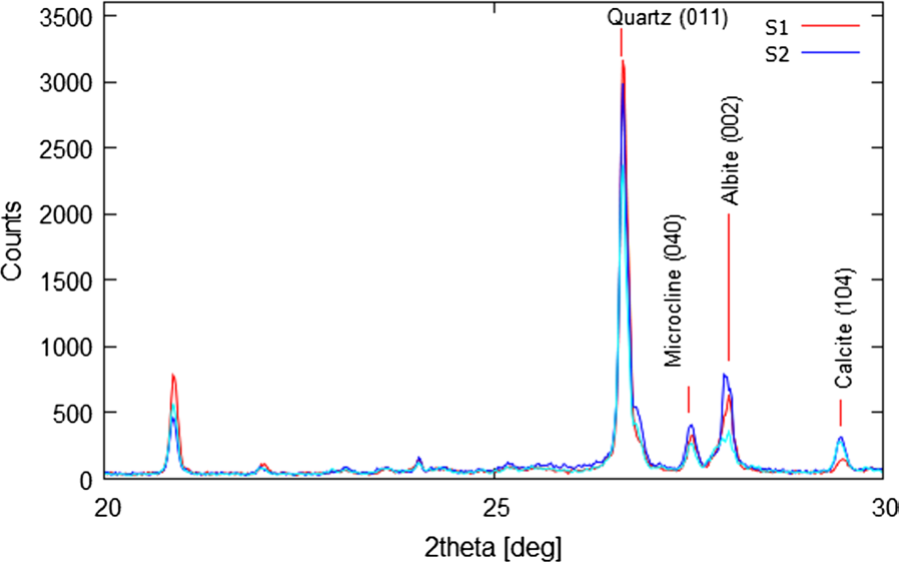
Diffraction patterns of soil samples S1 (set 1) and S2 (set 2).

The XRD pattern of the soil samples of two sets coded with S1 and S2 (Figure 5) of studied area showed their constituent phases to be almost similar even though in different relative amount: quartz, plagioclase and K feldspars, chlorites, calcite, amphiboles and albite. However in the case of clay minerals (<2 µm) identification, a different result obtained for soil samples S1 and S2, which look like bentonitic soils: quartz, montmorillonite, plagioclase and zeolites are their main constituents.

**Figure 6:**
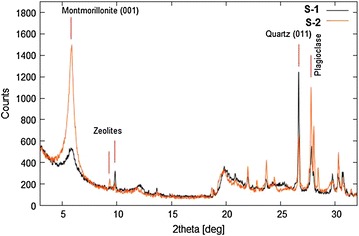
Diffraction pattern of clays minerals present in soil samples S1 (set 1) and S2 (set 2).

As apparent from Figure 6, the montmorillonite crystallinity for S2 is higher than S1. Moreover the increased intensity of peaks between 2θ = 27 and 29 degree suggests a higher presence of fresh (poor-altered) plagioclase. After matching the phases for S2, plagioclase and zeolite are represented by anorthite and laumontite, however they are only indicative of their silicate family. In fact it is likely that in plagioclase, there is an isomorphism mixture of phases ranging from two end-members anorthite to albite, Ca-rich and Na-rich plagioclase. Moreover, the simultaneous occurrence of well-crystallized montmorillonite, zeolites and fresh plagioclase with a significant anorthite component suggests an origin from volcanic glass and a relatively low transport of soils.

The presence of Ca-rich and Na-rich plagioclase and Ca-montmorillonite mineral in the soil might be responsible for the high sorption of uranium due to exchange with Ca^2+^ ions in the mineral lattice. Generally, sorption of the uranium is known to take place primarily as an exchange reaction with metal ions, particularly Ca^2+^, Na^+^ and K^+^ present in the clay minerals such as Ca -montmorillonite, Na-montmorillonite, illite respectively. Zeolites are also known for their high adsorption capacity for many cations, however, is unable to adsorb relatively high concentration of uranium.

### Chemical analyses

The soils were further characterized with regard to chemical composition in the form of major, trace metals and non metal as given in Table 2. The mean content of major elements as K, Ca and Fe in soil was observed to be 2.74, 4.01 and 3.77% respectively. However, trace elements as Cu, Ni, Co and Mn estimated to be 17, 18, 21 and 410 mg kg^−1^ respectively. Similarly, HA (Aldrich) was also analyzed for N, C, H and S to check the purity and estimated to be 2.47, 40.61, 2.98 and 1.28% respectively. The reason for relatively higher sorption of U in soil of set-2 could be also due to high abundances of total C content leading to strong complexation processes and high Fe content. In general, Fe is precipitated as oxy-hydroxide under alkaline pH which has the high affinity to scavenge other metals. On the contrary, low abundance of Ca particularly in set 2 exhibited high sorption confirming that U might be participated in the cation exchange processes among Ca-bearing minerals.

**Table 2:** Chemical composition of soil

Soil	K (%)	Ca (%)	Fe (%)	Cu (mg kg^−1^)	Ni (mg kg^−1^)	Co (mg kg^−1^)	Mn (mg kg^−1^)	N (%)	C (%)	H (%)	S (mg kg^−1^)
Set-1	2.24	4.67	2.87	12	26	27	435	1.74	0.68	0.21	150
Set-2	3.24	3.45	4.68	22	10	15	385	0.80	1.10	0.18	220

## Conclusions

The sorption of U onto soils increases at its low concentration range thereafter decreases at sufficiently high concentration range. Furthermore, the relatively higher sorption of U onto soil might be also affected by high abundances of finer particles in the form of clays. Results also indicated that sorption is strongly dependent on kind of clay minerals, temperature and presence of HA. The thermodynamic data suggested that the sorption reaction is spontaneous and endothermic. The HA appears to be a key-component when the objective of the study is to assess the potential mobility of U in natural systems. It is also suggested that the migration of uranium in soils in the presence of HA can be either accelerated by formation of the humic compounds or partly retarded by sorption of humic compounds. This study also reveals the susceptibility of U toxicity depending on sorption capacity of soil.
